# *In vivo* liver targeted genome editing as therapeutic approach: progresses and challenges

**DOI:** 10.3389/fgeed.2024.1458037

**Published:** 2024-08-23

**Authors:** Chiara Simoni, Elena Barbon, Andrés F. Muro, Alessio Cantore

**Affiliations:** ^1^ San Raffaele Telethon Institute for Gene Therapy (SR-Tiget), IRCCS San Raffaele Scientific Institute, Milan, Italy; ^2^ Vita-Salute San Raffaele University, Milan, Italy; ^3^ International Center for Genetic Engineering and Biotechnology, Trieste, Italy

**Keywords:** liver, genome editing, liver directed genome editing, delivery methods, lipid nano particle, viral vectors

## Abstract

The liver is an essential organ of the body that performs several vital functions, including the metabolism of biomolecules, foreign substances, and toxins, and the production of plasma proteins, such as coagulation factors. There are hundreds of genetic disorders affecting liver functions and, for many of them, the only curative option is orthotopic liver transplantation, which nevertheless entails many risks and long-term complications. Some peculiar features of the liver, such as its large blood flow supply and the tolerogenic immune environment, make it an attractive target for *in vivo* gene therapy approaches. In recent years, several genome-editing tools mainly based on the clustered regularly interspaced short palindromic repeats associated protein 9 (CRISPR-Cas9) system have been successfully exploited in the context of liver-directed preclinical or clinical therapeutic applications. These include gene knock-out, knock-in, activation, interference, or base and prime editing approaches. Despite many achievements, important challenges still need to be addressed to broaden clinical applications, such as the optimization of the delivery methods, the improvement of the editing efficiency, and the risk of on-target or off-target unwanted effects and chromosomal rearrangements. In this review, we highlight the latest progress in the development of *in vivo* liver-targeted genome editing approaches for the treatment of genetic disorders. We describe the technological advancements that are currently under investigation, the challenges to overcome for clinical applicability, and the future perspectives of this technology.

## Introduction

**FIGURE 1:**
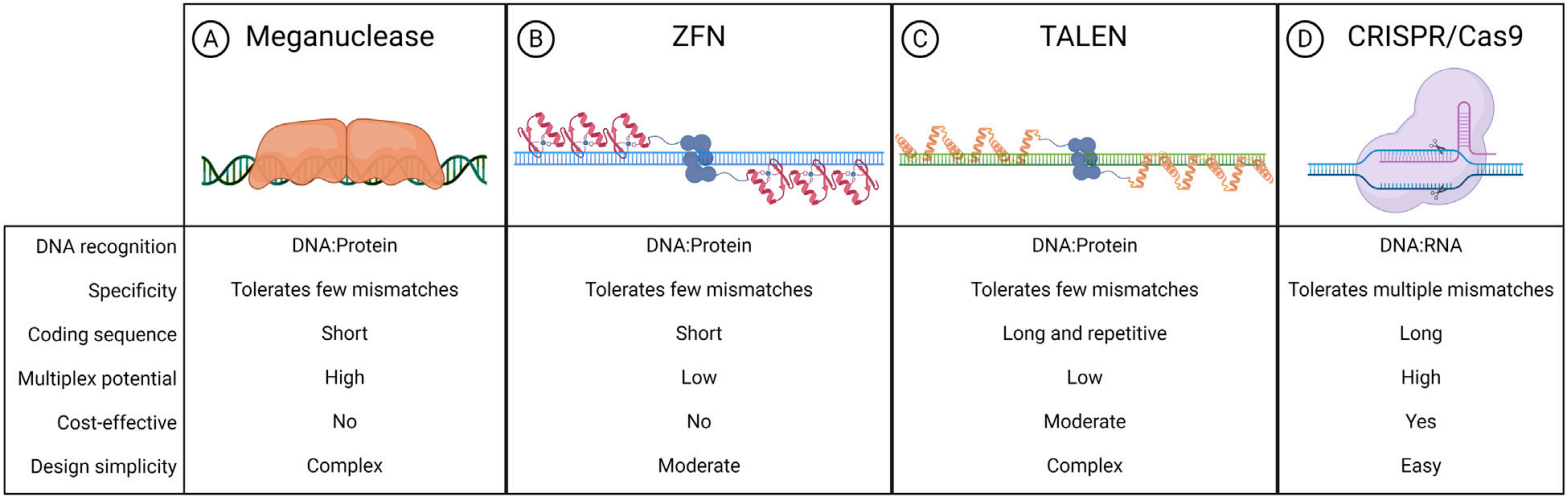
Nucleases exploited for genome editing. Schematic representation of meganuclease **(A)**, zinc finger nuclease (ZFN) **(B)**, transcription activator-like effector nuclease (TALEN) **(C)**, clustered regularly interspaced short palindromic repeats associated protein 9 (CRISPR-Cas9) **(D)**, and their respective features. Created with BioRender.com.

**FIGURE 2:**
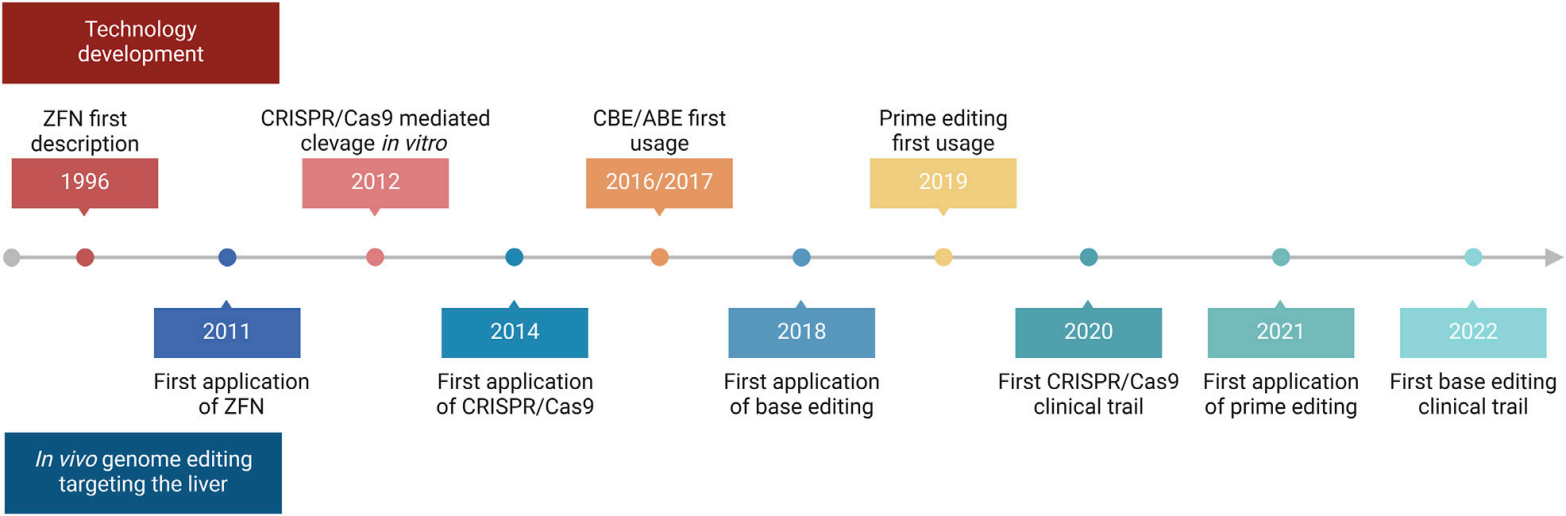
Timeline of *in vivo* liver-directed genome editing. Timeline chart highlighting crucial events regarding technological advancement and applications of *in vivo* liver-directed genome editing. Created with BioRender.com.

The liver is a major metabolic organ that performs around 500 essential biological functions, among them carbohydrate, fat, and protein metabolism, and the production and secretion of bile and coagulation factors. For this reason, impairment of liver function is the cause of many genetic diseases (Trefts et al., 2017). For some of these disorders, the restoration of the missing function relies on enzyme replacement therapy or orthotopic liver transplantation. Enzyme replacement therapy requires life-long repeated administrations of the therapeutic protein and can be performed only when the infused enzyme works in the blood, such as in plasma protein deficiencies, or can be uptaken by the target cells allowing cross-correction, such as in the case of lysosomal storage disorders (Bonam et al., 2019). On the other hand, liver transplantation is an invasive treatment that presents a risk of organ rejection and graft failure and requires a life-long immunosuppressive regimen (Khalil et al., 2023). For these reasons, in the past few decades, liver-directed gene therapy has been investigated as a promising alternative therapeutic strategy. To date, the most advanced approach is based on gene addition exploiting adeno-associated viral vectors (AAVs) injected systemically (Baruteau et al., 2024). AAVs targeting the liver to replace coagulation factors have been shown to provide multi-year benefit in hemophilic patients, leading to the approval of three gene therapy products, Etranacogene Dezaparvovec and Fidanacogene Elaparvovec for the treatment of hemophilia B (Heo, 2023; Pfizer, 2024) and Valoctocogene Roxaparvovec for the treatment of hemophilia A (Blair, 2022; Ozelo et al., 2022). Despite these achievements and promising results from several AAV-based therapeutic approaches that are currently being tested in pre-clinical and clinical studies, some limitations remain to the usage of AAVs (Wang D. et al., 2019). One major limitation is represented by the non-integrative nature of the virus which remains mainly episomal, thus leading to a progressive loss of transgene expression following cell replication (Cunningham et al., 2008; Wang et al., 2012). This would represent a challenge for the treatment of pediatric patients in which the liver is actively growing, especially in the context of severe metabolic disorders, where an early intervention would be essential to prevent disease progression and related complications (Yilmaz et al., 2020). To overcome this limitation, a possibility is to exploit integrative vectors such as lentiviral vectors (LVs), which have been shown to provide long-lasting transgene expression from the liver in hemophilia mouse models, dogs, and non-human primates (NHPs) (Cantore et al., 2015; Milani et al., 2019; Milani et al., 2022). Other strategies aiming at guaranteeing a stable transgene expression throughout post-natal liver growth and homeostatic turnover rely on transposon-based integration of a therapeutic cassette (Kebriaei et al., 2017). For both LV and transposon-based platforms, however, the semi-random integration profile and the usage of heterologous promoters cause concerns about genotoxicity. Moreover, employing constitutive promoters may not be ideal for applications in which a regulated transgene expression is preferred, such as when the overexpression of a protein could be detrimental to the cell, or when the gene expression is tightly regulated in response to specific stimuli. In this context, genome editing approaches are being increasingly explored to provide precise genomic interventions such as gene disruption, insertion of a therapeutic gene in a desired locus, correction of a specific disease-causing mutation, gene silencing, or gene activation (Adlat et al., 2023). The first reported genome editing tools were meganucleases, microbial-derived homing endonucleases adapted for targeted DNA modification (Figure 1A) (Stoddard, 2011). Next, synthetic fusion proteins called zinc finger nucleases (ZFNs) have been established by combining multiple zinc finger DNA binding domains with the catalytic domain of FokI, a restriction enzyme that cleaves the double-strand DNA (Figure 1B) (Urnov et al., 2010). Finally, the same FokI domain has also been exploited in combination with transcription activator-like effector proteins to generate transcription activator-like effector nucleases (TALENs) (Figure 1C) (Christian et al., 2010). However, redirecting the DNA targeting specificity of these genome editing tools based on protein-DNA interactions requires laborious steps of protein engineering, thus these reagents never gained widespread usage in the field. By contrast, most genome editing approaches nowadays rely on the usage of Clustered Regularly Interspaced Short Palindromic Repeats and associated protein 9 (CRISPR/Cas9), which has become over the years the system of choice, thanks to the fact that targeting specificity is based on RNA-DNA complementarity (Figure 1D) (Wang and Doudna, 2023). A ribonucleoprotein (RNP) composed of a simple and short single-guide RNA (sgRNA) and the Cas9 nuclease can indeed allow targeting a wide range of DNA sequences (Wang and Doudna, 2023). CRISPR/Cas9 is a family of proteins divided into two classes, each further divided into three types and several subtypes (Makarova et al., 2020). The most exploited is class 2 since the proteins involved are smaller than those in class 1. CRISPR/Cas class 2 system can be divided into type II/Cas9, type V/Cas12, and type VI/Cas13. The target of Cas9 and Cas12 is double-strand DNA while Cas13 targets RNA. Cas9 and Cas12 differ in their mechanism of action and dimensions since Cas12 is smaller (∼3,600 nucleotides) and requires a shorter sgRNA compared to Cas9 (van der Oost and Patinios, 2023). The description of this mechanism by the work of Doudna and Charpentier was revolutionary for modern biotechnology and was indeed recognized with the Nobel Prize for chemistry in 2020 (Jinek et al., 2012). All meganucleases, TALENs, ZFNs, and Cas nucleases can thus be programmed to perform site-specific DNA double-strand breaks (DSBs). The DSBs can be repaired by exploiting different endogenous repair pathways such as the non-homologous end joining (NHEJ), the micro homology-mediated end joining (MMEJ), or the homology-directed repair (HDR) (Chen X. et al., 2024). It is possible to take advantage of all these cellular DNA repair mechanisms depending on the intended genomic modification. Since the presence of DBS can potentially give rise to genotoxicity concerns (Tao et al., 2023), the nucleases have also been engineered to perform a single-strand cut (i.e., nickase Cas9, nCas9), or to be catalytically inactive (i.e., dead Cas9, dCas9). These modified Cas9 have then been fused to deaminases, retro-transcriptases, transcriptional activators, or silencing proteins to obtain, theoretically, safer genomic or epigenomic modifications (Tao et al., 2023). All the rapid advances in this field have led to the approval of the first CRISPR/Cas9-based gene editing product, Casgevy, in December 2023 (FDA, 2023; Sheridan, 2024). The drug is based on the *ex vivo* modification of patients’ hematopoietic stem cells (HSCs) to disrupt the expression of *BCL11A*, a repressor of the fetal γ-globin gene, for the treatment of sickle cell disease and transfusion-dependent β-thalassemia. As a result of the *BCL11A* inactivation, the reactivation of fetal hemoglobin allows for restoring erythropoietic homeostasis (Frangoul et al., 2021). Treatments for various other diseases are currently under investigation exploiting the *ex vivo* modification of HSCs. However, genome editing is more difficult to apply in the context of diseases linked to solid organs like the liver (Baruteau et al., 2024), which require an *in situ* delivery of the editing machinery. Nevertheless, in recent years great improvements have been performed opening the possibility for liver-directed genome editing to become a treatment for inherited and acquired diseases (Figure 2). In this review, we highlight the latest progress in the development of *in vivo* liver-targeted genome editing approaches for the treatment of genetic disorders, with a focus on the different strategies applied and the optimization of the delivery methods. We provide an overview of the technological advancements currently under investigation, focusing on recent pre-clinical and clinical research. We discuss the challenges to be overcome for clinical translation and future perspectives of this technology.

## Gene editing strategies

The technological advances in genome editing opened the way to many different approaches that can be exploited to treat liver genetic diseases. Simple gene disruption has been achieved by exploiting the NHEJ pathway, giving rise to insertions or deletions (indels) able to abrogate gene expression (Stinson and Loparo, 2021). On the other end, the different pathways of DSB repair (NHEJ, MMEJ, and HDR) have been exploited to obtain the insertion of the transgene of interest, carried by a donor DNA, in the desired locus (Chen X. et al., 2024). More recently, base editing and prime editing have been developed to target specific disease-causing mutations with the advantage of greatly reducing the generation of DSB events (Anzalone et al., 2020). Lastly, some strategies have been developed that completely avoid breaks of DNA, based on the modification of the epigenome to transcriptionally silence or activate a specific gene (Villiger et al., 2024). In the following subsections, we reviewed representative works on the above-mentioned approaches, which are also listed in Table 1.

**TABLE 1:** Non-exhaustive representative list of studies employing genome editing therapeutic approaches targeting the liver.

Genome editing strategy	Genomic target	Disease indication	Delivery method	References
NHEJ-mediated gene disruption	*TTR*	Transthyretin Amyloidosis	CRISPR/Cas9 delivered by LNPs	Finn et al. (2018), Gillmore et al. (2021)
	*KLKB1*	Hereditary Angioedema		Longhurst et al. (2024)
Homology-independent targeted integration strategy (HITI)	*ALB*	Mucopolysaccharidosis type VI	CRISPR/Cas9 and donor DNA delivered by AAVs	Tornabene et al. (2022), Esposito et al. (2024)
		Hemophilia A, B		Chen et al. (2019), He et al. (2022), Esposito et al. (2024)
	*SERPINC1*	Hemophilia A, B	CRISPR/Cas9 and donor DNA delivered by LNPs + AAVs	Han et al. (2023), Lee et al. (2023)
MMEJ-mediated gene disruption	*HAO1*	Primary Hyperoxaluria type 1	CRISPR-Cas9 nickases delivered by AAVs	Torella et al. (2024)
MMEJ-mediated gene insertion	*FAH*	Hereditary tyrosinemia type 1	CRISPR-Cas9 plasmids by hydrodynamic injection	Yao et al. (2017)
HDR-mediated gene insertion (without nucleases)	*ALB*	Hemophilia B, Crigler-Najjar, methylmalonic acidemia, Wilson disease	Promoterless donor DNA delivered by AAVs	Barzel et al. (2015), Porro et al. (2017), Chandler et al. (2021), Venturoni et al. (2022), Padula et al. (2023)
HDR-mediated gene insertion (with nucleases)	*F9*	Hemophilia B, Crigler Najjar	CRISPR/Cas9 and donor DNA delivered by AAVs + LNPs, or dual AAVs	Wang et al. (2019b), Lisjak et al. (2022)
	*ALB*			De Caneva et al. (2019)
	*PAH*	Phenylketonuria		Richards et al. (2020)
	*SPF*	Ornithine transcarbamylase deficiency		Wang et al. (2020)
	*ASS1*	Citrullinemia type I		Lisjak et al. (2023)
	*F9*	Hemophilia B	ZFN and donor DNA delivered by AAVs	Li et al. (2011), Sharma et al. (2015)
	*IDUA*	Mucopolysaccharidosis type I		Ou et al. (2019)
	*GLA*	Fabry disease		Pagant et al. (2021)
Base editing-mediated gene correction	*PAH*	Phenylketonuria	ABE reagents delivered by LNPs	Brooks et al. (2023), Brooks et al. (2024)
	*IDUA*	Mucopolysaccharidosis type I	ABE delivered by dual AAVs	Su et al. (2023)
	*HFE*	Hemochromatosis		Rovai et al. (2022)
	*AGXT*	Primary Hyperoxaluria type 1		Chen et al. (2024b)
	*PAH*	Phenylketonuria		Villiger et al. (2018)
	*SERPINA1*	Alpha-1 antitrypsin deficiency	ABE or CBE reagents delivered by LNPs	Packer et al. (2022)
Base editing-mediated gene disruption	*PCSK9*	Familiar hypercholesterolemia	ABE reagents delivered by LNP	Musunuru et al. (2021), Rothgangl et al. (2021)
Prime editing-mediated gene correction	*SERPINA1*	Alpha-1 antitrypsin deficiency	PE delivered by dual AAVs	Liu et al. (2021)
	*PAH*	Phenylketonuria	PE delivered by AdV	Böck et al. (2022)
	*FAH*	Hereditary tyrosinemia type 1	PE-plasmids delivered by hydrodynamic injection	Jang et al. (2021)
	*PCSK9*	Coronary diseases	PE delivered by dual AAVs	Zheng et al. (2022), Davis et al. (2024)
Prime editing-based deletion and replacement of long DNA sequences	*FAH*	Hereditary tyrosinemia type 1	PE-plasmids delivered by hydrodynamic injection	Jiang et al. (2022), Zheng et al. (2023)
	*ACTB*	/	PE delivered by AdV	Yarnall et al. (2023)
Epigenome editing-mediated gene activation	*EPO*	Chronic renal anemia	Epigenome editors delivered by LNPs	Beyersdorf et al. (2022)
Epigenome editing-mediated gene repression	*PCSK9*	Coronary diseases	Epigenome editors delivered by dual AAVs	Thakore et al. (2018), Gemberling et al. (2021)
			Epigenome editors delivered by LNPs	Cappelluti et al. (2024)

### NHEJ-based gene editing approaches

**FIGURE 3:**
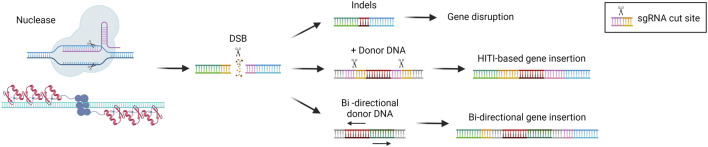
Non-homologous end joining (NHEJ)-based gene editing strategies. Scheme of NHEJ-based gene editing approaches and their possible outcomes, i.e., gene disruption *via* indels formation or gene insertion in the presence of a donor DNA (HITI-based or bi-directional). Created with BioRender.com.

NHEJ is the DSB repair pathway most exploited by the cells and is active in all phases of the cell cycle (Pannunzio et al., 2018). During the repair process, DNA-free ends are processed to obtain blunt ends that are directly ligated. This system is therefore prone to the introduction of indel mutations since there are no mechanisms of DNA proofreading (Stinson and Loparo, 2021) (Figure 3). For this reason, NHEJ is typically exploited to target monogenic disorders caused by a gain-of-function or dominant negative mutations, by inactivating the target gene (Schambach et al., 2024). One of the most advanced approaches is the one developed for the treatment of Transthyretin Amyloidosis, an autosomal dominant condition resulting from mutations in the *TTR* gene, leading to transthyretin accumulation in tissues. The strategy developed exploits CRISPR/Cas9 and the NHEJ pathway to disrupt the *TTR* gene in hepatocytes (Finn et al., 2018; Gillmore et al., 2021), with promising clinical efficacy (see “Liver-directed genome editing clinical trials” section below). A similar strategy has been developed for the treatment of Hereditary Angioedema, a dominant genetic disorder due to C1-inhibitor protein overexpression. In this case, a CRISPR/Cas9-based strategy aims at disrupting the *KLKB1* gene, which is involved in the overproduction of the C1-INH protein (Longhurst et al., 2024). Both strategies have shown to be effective and are now being tested in clinical trials (see “Liver-directed genome editing clinical trials” section below). The NHEJ pathway has also been exploited to achieve targeted integration of a therapeutic transgene. In 2016 Suzuki et al. first described the homology-independent targeted integration strategy (HITI) (Suzuki et al., 2016), based on the usage of a donor DNA flanked by the sgRNA target sequences in reverse orientation. In this way, Cas9 can cut both the genomic target locus and the donor DNA to improve the genomic integration rate. Moreover, if the genomic insertion occurs in the wrong orientation, an intact sgRNA target sequence may be recreated and re-cut, thus favoring donor integration in the proper orientation (Suzuki et al., 2016) (Figure 3). This strategy has been investigated in a preclinical study for the treatment of the lysosomal storage disease Mucopolysaccharidosis type VI. It resulted in therapeutic efficiency and stable integration in the liver of mice treated as newborns (Tornabene et al., 2022). The HITI strategy has been applied also in the context of hemophilia A and B, caused by mutations in coagulation factor 8 or 9 (*F8* or *F9*) respectively. In recent studies, the corrective donor DNA was inserted in the albumin locus, to exploit the strength of the albumin promoter and achieve high expression of the coagulation factor transgene and its secretion into the bloodstream (He et al., 2022). The power of HITI-mediated gene insertion for disease treatment has been confirmed recently in another preclinical study for Mucopolysaccharidosis type VI and hemophilia A (Esposito et al., 2024). The targeted integration of a transgene in the albumin locus is particularly beneficial in the context of non-autonomous cell disorders or disorders in which the organism can be detoxified by cross-correction mechanisms. In this way, even a relatively low targeting efficiency paralleled by a low percentage of corrected hepatocytes may guarantee therapeutic levels of transgene expression thanks to the strong activity of the albumin promoter. Another strategy tested in the context of hemophilia consisted of the integration of the corrective complementary DNA (cDNA) into the *Serpinc1* locus. The integration was based on a bidirectional construct containing the *F8* or *F9* cDNA. In this way, the transgene can always be expressed, despite the orientation of the insertion (Han et al., 2023; Lee et al., 2023) (Figure 3). An additional potential application of NHEJ-mediated gene disruption is the treatment of hepatitis B virus (HBV) infection, which can lead to liver cirrhosis and cancer (Yuen et al., 2018). Recently, some strategies have been developed to disrupt essential genes of the virus Kasianchuk et al. (2023), showing a reduction of viral proteins in both serum and liver of HBV-infected mice (Yan et al., 2021). However, this approach still requires optimization to further reduce viral load and HBV DNA in CRISPR-treated mice (Stone et al., 2021).

### MMEJ-based gene editing approaches

**FIGURE 4:**
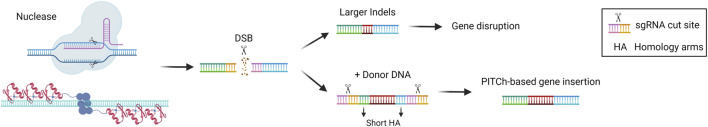
Microhomology-mediated end joining (MMEJ)-based gene editing strategies. Scheme of MMEJ-based gene editing approaches and their possible outcomes, i.e., gene disruption *via* larger indels formation or gene insertion in the presence of a donor DNA (PITCh). Created with BioRender.com.

MMEJ is an alternative pathway for the repair of DSB, which exploits 5–25 base pairs (bp)-long micro homologous sequences present in the two broken DNA strands. This pathway results in deletions or insertions that are longer compared to those usually introduced by NHEJ. Moreover, MMEJ is more active during the S/G2 phase of the cell cycle and seems to compete with NHEJ in the G1 phase (Sallmyr and Tomkinson, 2018). Given the fact that the MMEJ pathway has mutagenic properties, it has been exploited for therapeutic gene disruption purposes (Figure 4). A recent application has been described for the treatment of primary hyperoxaluria type 1, an autosomal recessive metabolic disease caused by mutations in the *AGXT* gene and leading to oxalate accumulation in the liver and kidney, leading to renal dysfunction and ultimately kidney failure. Two paired nCas9 were employed to introduce nicks on the two opposite strands of the *HAO1* gene, at a distance of 28 nucleotides. The nicks were repaired mainly by MMEJ and led to the introduction of indels that abolished the gene function, resulting in therapeutic efficacy in a disease mouse model (Torella et al., 2024). MMEJ can be also exploited for targeted genomic integration. In a strategy called PITCh (precise integration into target chromosome), micro-homology arms flank the donor DNA allowing for the genomic integration mediated by the MMEJ pathway. As for the HITI strategy, sgRNA target sequences can be added at the side of the donor DNA to increase the rate of insertion (Nakade et al., 2014) (Figure 4). PITCh strategy has been tested by injection of the MMEJ components into the liver of mice with the metabolic disease hereditary tyrosinemia type I, to insert a *Fah* therapeutic cDNA into the endogenous genomic locus. The strategy resulted in reduced liver damage and a better survival rate of the treated animals, indicating the rescue of *Fah* expression from those hepatocytes in which a functional integration occurred (Yao et al., 2017).

### HDR-based targeted integration approaches

**FIGURE 5:**
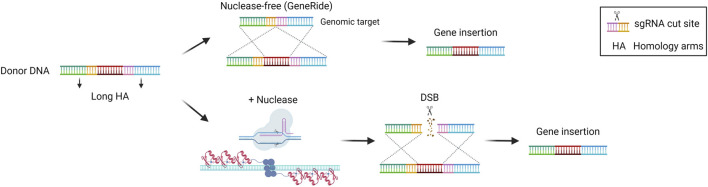
Homology-directed repair (HDR)-based gene editing strategies. Scheme of HDR-mediated gene insertion mechanisms, achieved *via* recombination with or without nucleases. Created with BioRender.com.

HDR is the most precise mechanism that cells exploit to repair DSB in the genome. It relies on the presence of the sister chromatid, so it is active during the S/G2 phase of the cell cycle. It involves the recognition, protection, and processing of the DNA ends, that are paired with the homologous regions. Subsequentially, DNA polymerase can synthesize the complementary sequence that is missing. The two complementary regions are then annealed, processed, and ligated to restore the intact genome (Douglass Wright et al., 2018). This precise repair pathway is the most commonly exploited in the genome editing field to allow for the precise insertion of sequences of interest in a site-specific manner. It has been demonstrated that the HDR pathway can be exploited even without nucleases to perform gene targeting. Several studies highlighted the potential of inserting a promoterless therapeutic cDNA into the albumin locus as a safe harbor to exploit the high activity of the albumin promoter for transgene expression (Barzel et al., 2015; Porro et al., 2017). In particular, in the approach named GeneRide, the cDNA of interest is inserted in-frame into the 3′ end of the albumin gene along with an upstream 2A-peptide coding sequence, to allow for the translation of both the albumin-2A peptide and the therapeutic protein (Figure 5). This approach has been tested in pre-clinical proof of concept studies for the treatment of hemophilia B and Crigler-Najjar syndrome, resulting in therapeutic efficacy even at low targeting efficiencies, since in these disorders even a relatively low enzyme activity results in clinical benefit (Barzel et al., 2015; Porro et al., 2017). The nuclease-free strategy has been investigated also in the context of Wilson disease, an autosomal disorder of copper accumulation caused by mutations in the *ATP7B* gene, encoding a transporter involved in copper excretion into the bile. A promoterless mini-*ATP7B* transgene inserted in the albumin locus resulted in extensive liver repopulation by edited hepatocytes and amelioration of liver injury and copper metabolism (Padula et al., 2023). The same strategy has also been tested in the context of methylmalonic acidemia, a severe organic acidemia caused by a mitochondrial enzymatic defect. In this case, the initial low targeting efficiency was compensated over time by a progressive expansion of the corrected cells harboring a selective advantage over non-corrected ones, with a concomitant amelioration of the disease-related biomarkers (Chandler et al., 2021; Venturoni et al., 2022). These results supported the initiation of a phase I/II clinical trial in pediatric patients with severe methylmalonic acidemia sponsored by LogicBio Therapeutics, which however is now terminated due to lack of efficacy and the occurrence of severe adverse events (SAEs) in some patients (ClinicalTrials, 2024a; Payton et al., 2024). Efforts have been made to increase the targeting efficiency. It has been demonstrated that fludarabine, a ribonucleotide reductase inhibitor, could increase *in vivo* nuclease-free HDR efficiency by around 3 to 4-fold in the murine liver (Tsuji et al., 2022). Nevertheless, the nuclease-free strategy is difficult to apply to most diseases where there is no proliferative advantage of the corrected hepatocytes or in which an initial high targeting efficiency is required to prevent disease-related complications. For this reason, HDR-based donor DNA is usually coupled with a nuclease such as Cas9, to generate a DSB in the locus of interest favoring the HDR-mediated integration rate (Figure 5) (Rouet et al., 1994). This strategy has been applied in the context of hemophilia B, to insert a corrective *F9* cDNA into the second exon of the *F9* gene using ZFN or CRISPR/Cas9 (Li et al., 2011; Sharma et al., 2015; Wang L. et al., 2019) or the 3′end of the albumin safe harbor (Lisjak et al., 2022). In the first approach, ZFN were used to target adult mice with an *F9* cDNA bearing a splice site acceptor upstream of the coding region. On the other hand, Wang et al. delivered the CRISPR/Cas9 machinery in both newborn and adult hemophilia B mice, showing around 10% and 4% of hepatocyte targeting by HDR respectively, thus resulting in stable FIX expression even after partial hepatectomy (Wang L. et al., 2019). In the second approach, Lisjak et al. also delivered the CRISPR/Cas9 components and the donor DNA, confirming a higher HDR efficiency in the liver of newborn mice (around 4%) compared to adults (less than 1%). This resulted in therapeutic efficacy only in mice treated as newborns (Lisjak et al., 2022), highlighting the challenge of performing efficient HDR-targeting in the post-mitotic adult liver in which NHEJ is preferentially exploited compared to HDR. The potential of HDR-based targeting coupled with a nuclease was demonstrated also in other preclinical studies aiming at integrating the donor DNA into the endogenous genomic locus or in a safe harbor, in the context of inherited metabolic diseases, such as phenylketonuria (Richards et al., 2020), ornithine transcarbamylase deficiency (Wang et al., 2020), citrullinemia type I (Lisjak et al., 2023), and Crigler-Najjar syndrome (De Caneva et al., 2019). These studies confirmed a generally higher efficiency of integration when using the nuclease compared to the GeneRide approach. Not only Cas9 but also ZFNs were employed coupled with the donor DNA for site-specific insertion of a corrective transgene into the hepatocyte albumin locus, resulting in some therapeutic efficacy in mouse models of the lysosomal storage diseases mucopolysaccharidosis type I, Gaucher, Hurler, and Fabry disease (Sharma et al., 2015; Ou et al., 2019; Pagant et al., 2021).

### Liver-targeted base editing approaches

**FIGURE 6:**
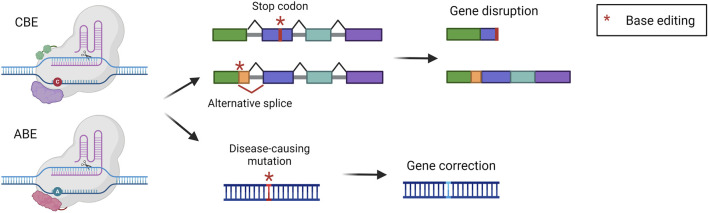
Base editing strategies. Scheme of base editing approaches and their possible outcomes, i.e., gene disruption *via* stop codon or alternative splice formation, or gene correction of a point mutation. Created with BioRender.com.

Base editing is an innovative technology that has been developed by the group of David Liu to perform precise nucleotide modification without the need for potentially harmful DSB (Komor et al., 2016; Gaudelli et al., 2017). Base editors (BEs) are composed of nCas9 fused to a deaminase enzyme, which catalyzes the deamination of a nucleobase. The strategy relies on the positioning of the deaminase on the target locus by the sgRNA and Cas9. Once Cas9 binds the genome the double-stranded DNA is open so that the deaminase can act precisely on a short stretch of single-stranded DNA (ssDNA), named the editing window. Modified nucleobases create a mismatch in the genome that is then solved by cellular DNA repair mechanisms. Concomitantly, the nCas9 cuts the non-modified strand favoring the usage of the other strand as a template for DNA repair, thus maintaining the edited nucleotide (Porto et al., 2020). The two most exploited and advanced editors are the cytidine base editor (CBE) and adenine base editor (ABE). CBE is designed to convert cytidine into uridine. This modification is read by the replication and transcription machinery as a thymidine, obtaining a C-G to T-A conversion. CBE has been derived from a natural cytidine deaminase coupled with Cas9 and with the uracil glycosylase inhibitor (UGI). UGI has been added to avoid the removal of the uracil, which occurs frequently since the base excision repair pathway is very active in cells (Komor et al., 2016). On the other hand, ABE was laboratory-evolved to act on DNA starting from RNA-targeting adenosine deaminases, then fused to nCas9. They can deaminate adenosine resulting in inosine, which is read by replication and transcription machinery as a guanine, resulting in an A-T to G-C base pair conversion (Gaudelli et al., 2017). BEs can be exploited to obtain different outputs. They can directly correct a disease-causing point mutation, or they can disrupt the expression of a gene. The disruption can be achieved by generating a premature stop codon or by creating an alternative splice site that will disrupt the original reading frame (Figure 6). The correction of a pathogenic mutation has been performed in the context of phenylketonuria, an autosomal recessive disorder caused by mutations in the *PAH* gene, leading to the accumulation of phenylalanine in the blood. A mouse model harboring one of the most common *PAH* pathogenic variants has been treated with an ABE, resulting in the normalization of circulating phenylalanine (Brooks et al., 2023; Brooks et al., 2024). ABE-mediated correction of pathogenic mutations was also assessed in preclinical studies for the treatment of mucopolysaccharidosis type I (Su et al., 2023), hemochromatosis (Rovai et al., 2022), and primary hyperoxaluria type 1 (Chen Z. et al., 2024). Furthermore, CBE was exploited for the treatment of phenylketonuria, restoring the physiological blood phenylalanine and ameliorating the disease phenotype (Villiger et al., 2018). Another work investigated two strategies for the treatment of alpha-1 antitrypsin deficiency. The first was based on the use of CBE to introduce a mutation with a compensatory functional effect, the second was based on the use of ABE to correct a disease-causing point mutation. Both strategies resulted in a therapeutically relevant increase in serum alpha-1 antitrypsin and improved liver histopathology (Packer et al., 2022). Besides genetic disorders, liver base editing has also been investigated for the treatment of hepatocellular carcinoma. In particular, Zhao et al. employed ABEs to correct a *TERT* promoter mutation, which frequently occurs in HCC and other human cancers. The correction of the mutation with an ABE resulted in significant inhibition of the growth of liver tumors in a mouse model (Zhao et al., 2024). BEs for gene disruption purposes have also been extensively investigated for the treatment of familial hypercholesterolemia, an autosomal dominant disease characterized by high circulating low-density lipoprotein (LDL) cholesterol (Canepari and Cantore, 2023). In 2021 two reports were published describing the usage of ABE for the disruption of a splice site, resulting in the knock-out of *PCSK9* in macaques. Since *PCSK9* is a negative modulator of the LDL receptor (LDLR), its inhibition resulted in LDLR upregulation in the liver, in turn leading to a therapeutic reduction of blood LDL cholesterol (Musunuru et al., 2021; Rothgangl et al., 2021). In 2023, these findings rapidly led to the initiation of a clinical trial in patients affected by familial hypercholesterolemia.

### Liver-targeted prime editing approaches

**FIGURE 7:**
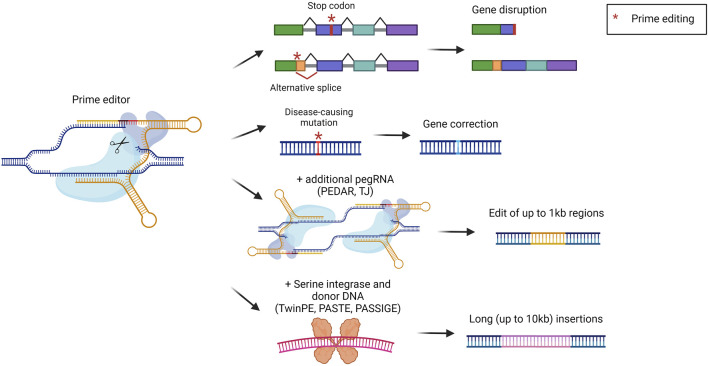
Prime editing strategies. Scheme of prime editing approaches and their possible outcomes, i.e., gene disruption *via* stop codon or alternative splice formation, gene correction, editing of up to 1 kb with two pegRNAs (PEDAR, TJ), or serine integrase-mediated long insertions (TwinPE, PASTE, PASSIGE). Created with BioRender.com.

Prime editors (PEs) represent one of the latest advances in the genome editing field. They were developed to broaden the possibilities for precise genomic modification that cannot be fully covered by BEs. PEs are composed of a nCas9 coupled with a reverse transcriptase (RT). The sgRNA has been modified to generate a prime editing gRNA (pegRNA). The pegRNA is similar to a standard sgRNA, with the addition of the primer binding site (PBS) and the RT template. The mechanism of action consists of the binding and nicking of the DNA template by Cas9. The cut DNA strand is then recognized by the pegRNA which anneals *via* the PBS. This annealing is exploited by the RT which retrotranscribes the template to install the desired DNA sequence. In the latest version of PEs, an additional sgRNA was added to introduce a nick in the non-edited strand, to favor the insertion of the modified DNA strand (Anzalone et al., 2019). PEs can be exploited to achieve short integrations, deletions, and all the possible base-to-base conversions (Figure 7). In comparison to standard editing with nucleases or with BEs, PEs display some advantages. For instance, they allow the minimization of DBS and modification of sequences far from the protospacer adjacent motif (PAM) (Anzalone et al., 2019). One of the first applications of PEs was related to the treatment of alpha-1 antitrypsin deficiency. The most frequent pathogenic mutation is a G to A mutation in the *SERPINA1* gene. In this genomic region, however, there is no optimal PAM to be used for editing. Therefore, Liu et al. investigated a prime editing strategy that resulted in the correction of around 7% of pathogenic alleles in the liver (Liu et al., 2021). Another study applied prime editing for the treatment of phenylketonuria, reaching a correction efficiency of around 11% in the liver of newborn mice, sufficient to provide therapeutic benefit (Böck et al., 2022). PEs were also delivered *in vivo* to the liver of mice with hereditary tyrosinemia, resulting in a significant amelioration of the disease phenotype without detectable off-target edits (Jang et al., 2021). Another relevant target for liver prime editing is *PCSK9*, as previously described for base editing strategies for the treatment of hypercholesterolemia. Thus, some works described the selection and optimization of efficient PEs to be delivered *in vivo* to target *PCSK9* (Zheng et al., 2022; Davis et al., 2024). Several studies have in common the continuous development of PEs to allow easier delivery to the liver and to increment the editing efficiency (Liu et al., 2021; Böck et al., 2022; Zheng et al., 2022; Davis et al., 2024). In this context, it would be beneficial to achieve integrations or deletions of large DNA fragments to correct defects arising from multiple mutations or large insertions/deletions. However, PEs can effectively insert only up to around 50 base pairs or delete up to around 80 base pairs (Anzalone et al., 2019). To address this issue different strategies have been developed, but few of them have already been tested *in vivo*. The strategy called PEDAR (PE-Cas9-based deletion and repair) exploits a fully active Cas9 combined with a retro transcriptase and two different pegRNAs (Figure 7). In this way, the region between the two pegRNAs can be deleted, since Cas9 will perform a DBS. Subsequently, the two pegRNAs will be retrotranscribed to form two complementary strands that will be ligated, resulting in the deletion of the undesired sequence and the insertion of a specific edit. PEDAR has been applied in a mouse model of tyrosinemia I leading to the deletion of 1.38 kilobases and the insertion of 19 base pairs (Jiang et al., 2022). A strategy tested *in vivo* for large DNA insertions is the so-called template jumping (TJ) prime editing, based on the presence of two primer-biding sites on the pegRNA. In this case, PEs act as previously described, creating a ssDNA harboring another PBS at the end. The PE can then generate another nick on the opposite strand, *via* the other sgRNA. The PBS present on the ssDNA can bind the DNA at the nick site and the retro transcriptase can generate the second strand of DNA. Thus, the synthesized DNA is inserted in the target locus, allowing the deletion of the sequence that was present between the two nicks (Figure 7). This strategy has been exploited in a mouse model of tyrosinemia I to replace an exon of the *Fah* gene. This resulted in 0.1% of corrected hepatocytes that acquired a proliferative advantage (Zheng et al., 2023). Other systems combine the activity of PEs with the ability of serine integrase to insert long sequences of DNA. Examples are TwinPE (twin prime editing) (Anzalone et al., 2022), PASTE (programmable addition via site-specific targeting elements) (Yarnall et al., 2023), and PASSIGE (prime-editing-assisted site-specific integrase gene editing) (Pandey et al., 2024). They exploit PEs to insert in a specific locus the landing sequence recognized by the serine integrase, which then can insert a double-strand donor DNA. All the systems reached more than 10 kb of cargo insertion cell lines and primary cells (Figure 7). PASSIGE is the latest evolution and allows the highest rate of integration *in vitro* (up to 60%) (Pandey et al., 2024). Among these approaches, PASTE was the only one tested *in vivo,* achieving around 1%–2% of integration in hepatocytes (Yarnall et al., 2023). Despite the great potential for precise genomic modification, prime editing needs to be optimized to increase efficiencies to be applied in different liver-related indications.

### Liver gene silencing/activation

**FIGURE 8:**
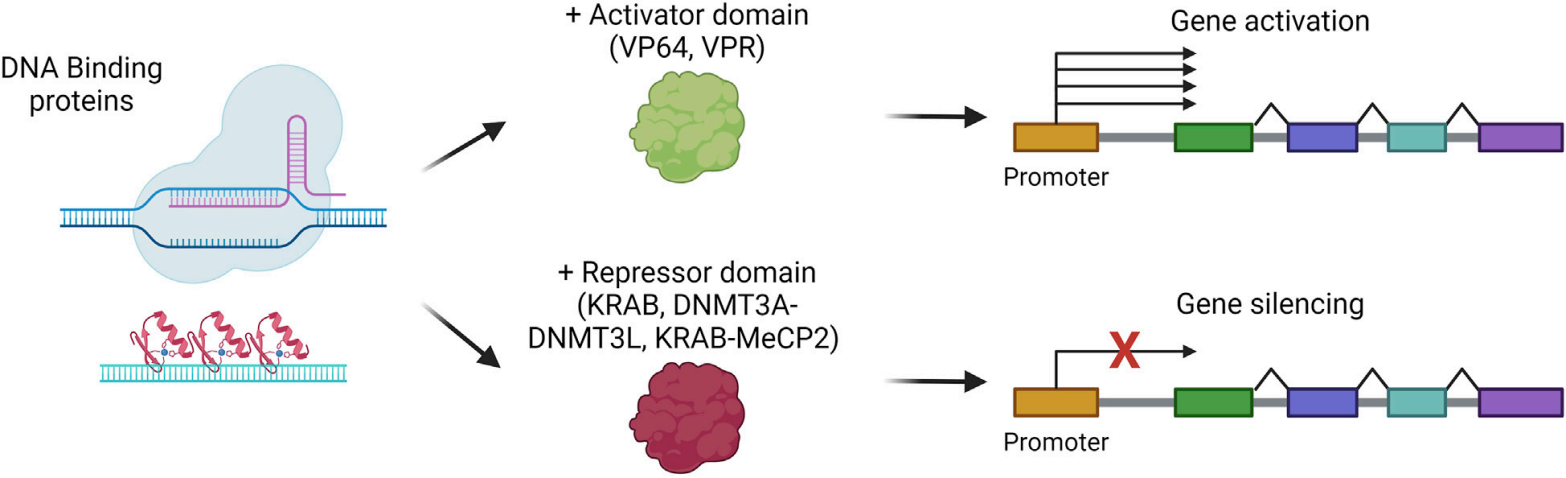
Gene silencing or activation strategies. Scheme of gene silencing or activation mechanisms, *via* the combination of DNA-binding proteins with repressor or activator domains, respectively. Created with BioRender.com.

Among the possible liver-directed therapeutic approaches, epigenome editing represents a strategy in active evolution for those indications that can benefit from gene silencing or gene activation. Indeed, epigenome editing aims at rewriting the epigenetic code to control gene expression without directly modifying the primary DNA sequence. Since epigenome editing does not intervene in disease-causing mutations, its application is limited to specific indications, for instance, to suppress the expression of a gene involved in a dominant genetic disease. The epigenome editors are based on proteins that can recognize and bind a specific region of the genome, like multiple zinc finger domains, transcription activator-like enhancer proteins, or dCas9, coupled to epigenome modifier enzymes (Ueda et al., 2023). The most commonly exploited epigenome activators are VP64 or the combination VP64-p65-Rta (VPR), while the most used repressors are KRAB, DNMT3A with DNMT3L, or the combination KRAB-MeCP2 (Amabile et al., 2016; Bendixen et al., 2023) (Figure 8). For their easy-to-assemble and easy-to-use properties, the most employed strategies are based on dCas9 fused to an activator (CRISPRa) or a repressor for interference (CRISPRi) (Bendixen et al., 2023). The potency of CRISPRa *in vivo* in hepatocytes has been demonstrated for the upregulation of erythropoietin, a target therapeutically relevant for several conditions. CRISPRa was delivered *in vivo*, obtaining sustained overexpression of erythropoietin up to 7 days (Beyersdorf et al., 2022). On the other hand, liver-targeted CRISPRi has been exploited in different works aimed at silencing *PCSK9* (Thakore et al., 2018; Gemberling et al., 2021). These studies showed a stable up-to-6-month reduction of the protein in the serum of treated mice. Of note, another recent work demonstrated that zinc-finger-based epigenome editors are outperforming CRISPR or TALE-based editors for the durable silencing of *PCSK9*, allowing for up to a 1-year reduction in circulating PCSK9, also after partial hepatectomy (Cappelluti et al., 2024). These works highlight the potential of epigenome editors for further development towards clinical applications.

## *In vivo* delivery methods to the liver

A crucial aspect to be considered when developing liver-directed genome editing approaches is the delivery of editing components *in vivo*. The editing machinery can be encoded by DNA or RNA templates, or it can be delivered directly as a protein or ribonucleoprotein (RNP) complex. All these macromolecules are fragile and can be easily degraded in circulation. Moreover, they need to reach the target tissue, interact with components of the plasma membrane or extracellular matrix, enter the cell, escape the endosome, and get to the appropriate intracellular compartment (Khirallah et al., 2023). For these reasons, optimal delivery vehicles should allow to efficiently encapsulate the editing components and then bring them to target cells, without toxicities for the recipient. This is a challenging endeavor, thus a significant effort has been put into developing different *in vivo* delivery strategies over the years. Delivery systems can be divided into viral and non-viral ones. Among the viral vectors, AAVs are the most utilized vehicles for genome editing applications *in vivo*, even if also LVs and adenoviruses-derived vectors (AdVs) could be considered as alternatives. Among non-viral methods, lipid nanoparticles (LNPs) are the most exploited and reached clinical application. Finally, virus-like particles (VLP) have recently been adopted in preclinical studies (Raguram et al., 2022).

### Adeno-associated viral vectors

**FIGURE 9:**
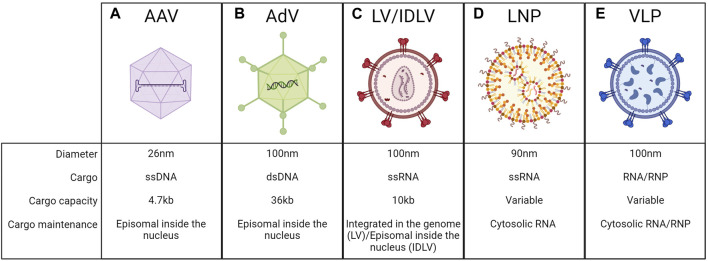
Delivery vehicles to the liver. Schematic representation of adeno-associated viral vectors (AAV) **(A)**, adenoviruses vectors (AdV) **(B)**, lentiviral vectors (LV) or integrase-deficient lentiviral vectors (IDLV) **(C)**, lipid nanoparticles (LNP) **(D)**, virus-like particles (VLP) **(E)**, and their respective features. Created with BioRender.com.

AAVs are derived from naturally occurring non-pathogenic viruses. They have an icosahedral capsid composed of 60 copies of structural proteins VP1, VP2, and VP3 with a diameter of ∼26 nm. Their genome is a ssDNA of ∼4.7 kb, flanked by two inverted terminal repeats (ITRs) that are essential for genome packaging during vector production (Wang D. et al., 2019). Once in the nucleus of the host cells, their genome remains mostly episomal, with a low rate of genomic integration (<1%), and it is therefore progressively diluted during cell replication (Cunningham et al., 2008; Wang D. et al., 2019) (Figure 9A). AAV-mediated liver gene transfer has shown promising efficacy and safety profile in several preclinical and clinical studies (Baruteau et al., 2024). Several native or engineered AAV-capsid variants have been exploited for specific applications, to reach different target cells (Pupo et al., 2022). For all these reasons they have become one of the most common methods also to deliver gene editing machinery. However, one limitation is their cargo capacity, limited to ∼4.7 kb. This is a relevant drawback for genome editing since the most exploited nuclease is the *Streptococcus pyogenes* Cas9 (SpCas9) which is ∼4 kb in length. Thus, the residual space available for regulatory elements and sgRNA expression cassettes is limited. Therefore, SpCas9-expressing AAVs typically contain very short promoters (up to 300 bp), whether constitutive (Suzuki et al., 2016; He et al., 2022) or liver-specific (Richards et al., 2020; Tornabene et al., 2022). Usually, a second AAV is needed for the delivery of the donor DNA and the sgRNA-expression cassette. In this case, an efficient genome editing output can be achieved only when the target cell is co-transduced by the two AAVs. The capacity of AAV to co-transduce the target cell was leveraged to develop a dual AAV vector strategy, in which a single gene product is split in half and then reconstituted upon co-transduction. The most successful dual AAV approach is based on protein trans-splicing using split inteins. The method was originally optimized for gene addition and gene replacement applications to deliver large transgenes (Trapani et al., 2014; Tornabene et al., 2019; Padula et al., 2022; Barbon et al., 2023; Jauze et al., 2024) and then applied also to deliver BEs, PEs, silencers, and activators for genome editing purposes (Levy et al., 2020; Davis et al., 2024). Upon co-transduction and transgene expression, the split inteins dimerize and undergo trans-splicing, generating the complete form of the transgene protein (Aranko et al., 2014). Dual AAV allowed successful base editing in the liver in the context of mucopolysaccharidosis type I (Su et al., 2023), hemochromatosis (Rovai et al., 2022), and phenylketonuria (Villiger et al., 2018). The dual AAV system was explored also for delivering PEs or epigenetic silencers in the context of alpha-1 antitrypsin deficiency (Liu et al., 2021), or to inactivate *PCSK9* expression (Thakore et al., 2018; Zheng et al., 2022). Since dual AAV approaches present efficiency, safety, and manufacturing-related limitations, different groups have tried to develop alternatives, such as smaller editing components. Smaller Cas9 orthologs or engineered Cas9 have been tested. The most exploited small Cas9 is derived from *Staphylococcus aureus* (SaCas9), which has a size of ∼3.2 kb SaCas9 has been successfully applied in preclinical works in the context of several inherited metabolic diseases and hemophilia (Wang L. et al., 2019; Chen et al., 2019; De Caneva et al., 2019; Wang et al., 2020; Lisjak et al., 2023; Torella et al., 2024). Recently, different laboratories developed smaller BEs and PEs that could potentially fit in a single vector (Davis et al., 2022; Zheng et al., 2022). Ideally, delivering the genome editing machinery by a single AAV would not only allow to achieve higher efficiency but also to potentially reduce the administered vector dose. Besides the cargo limitations, AAVs present additional challenges such as the persistent expression of the transgene in the transduced cells, if quiescent, which could increase the risk of unwanted off-target editing (Anzalone et al., 2020). Another risk is related to the potential immunogenicity of Cas9 and all the other components of the gene editing machinery which represent non-self-antigens (Wagner et al., 2021). This problem could be overcome by non-viral delivery methods, which we will discuss below.

### Adenoviral vectors and lentiviral vectors

AdVs are derived from adenoviruses, non-enveloped viruses with an icosahedral capsid of ∼100 nm in size. They have a linear double-stranded DNA genome of ∼36 kb, which is maintained as an episome in the nucleus of transduced cells (Lee et al., 2017) (Figure 9B). They were among the first vectors to be exploited for *in vivo* gene delivery, taking advantage of the knowledge of the biology of the parental virus and its capability of efficiently transducing the target cells (Lee et al., 2017). One of the most important advantages of AdV is the large cargo capacity, which has been exploited also for the delivery of genome editing machinery to the liver. They have been tested as an all-in-one platform for the delivery of Cas9 and sgRNA to knock out *PCSK9* (Ding et al., 2014), or together with another AdV carrying the donor DNA to allow insertion of the therapeutic transgene in the context of alpha-1-antitrypsin deficiency (Stephens et al., 2018) or hereditary tyrosinemia (Shao et al., 2018). AdV has been also explored to incorporate the coding sequences of BEs and PEs for *in vivo* delivery (Chadwick et al., 2017; Böck et al., 2022), as well as for the PASTE approach (Yarnall et al., 2023). However, the major concern related to the use of AdV is their immunogenic profile, which resulted in major adverse events in past gene therapy clinical trials employing these vectors (Raper et al., 2003). Moreover, AdV-derived Cas9 expression induced an anti-Cas9 immune response leading to the killing of corrected cells (Wang et al., 2015). For these reasons, the usage of AdV for *in vivo* genome editing will require further investigation before clinical testing. Another possible vehicle is represented by LVs, which are enveloped vectors derived from HIV, with a single-stranded RNA genome of ∼10 kb that is reverse-transcribed and integrated in a semi-random way into the genome of the transduced cells (Naldini et al., 1996) (Figure 9C). The most commonly used envelope protein is the vesicular stomatitis virus glycoprotein G (VSV.G), which makes LVs pantropic and allows the transduction of hepatocytes (Zufferey et al., 1997; Dull et al., 1998). To obtain a non-integrative vector, the integrase protein has been inactivated to generate integrase-deficient lentiviral vectors (IDLVs), so the viral genome after retro-transcription is maintained as an episome in the nucleus of the transduced cells (Wanisch and Yáñez-Muñoz, 2009) (Figure 9C). LVs have been exploited mainly for *ex vivo* applications and have already reached the clinic and the market for different indications (Labbé et al., 2021; Tucci et al., 2022). Their possible application for *in vivo* delivery to the liver has been investigated in different preclinical studies in dogs and NHP for hemophilia A and B (Cantore et al., 2015; Milani et al., 2019; Milani et al., 2022), and in mice for metabolic disorders (Barbon et al., 2024; Canepari et al., 2024). However, despite their cargo capacity could allow for relatively easy packaging of all the gene editing machinery in a single vector, they have not been used yet as an *in vivo* delivery of genome editing components to hepatocytes. In this regard, IDLVs have been demonstrated to be effective as HDR templates *ex vivo* (Lombardo et al., 2007) without raising concerns about integration-related genotoxicity. However, the risks related to the persistent expression of the editing agents and the off-target effects are the same of the AAVs.

### Lipid nanoparticles

LNPs have been developed in the past years for the *in vivo* delivery of short-interfering RNAs (siRNA) and messenger RNAs (mRNAs). Recently, they have been tested also for gene editing applications becoming one of the most exploited vehicles for liver targeting, for instance in clinical trials delivering genome editing reagents (Gillmore et al., 2021; Longhurst et al., 2024) (Figure 9D). LNPs are typically composed of four primary elements: phospholipids, cholesterol, polyethylene glycol (PEG) lipids, and ionizable cationic lipids (Kazemian et al., 2022). Phospholipids and cholesterol secure the structural stability of the LNP. PEG lipids have been introduced to coat LNP with PEG molecules to prevent particle aggregation, decrease immunogenicity, and increase the LNP half-life *in vivo* (Kazemian et al., 2022). Lastly, the use of ionizable cationic lipids has significantly improved the delivery mediated by LNP. Briefly, these lipids are positively charged during LNP production, allowing the interaction and encapsulation of negatively charged nucleic acids. On the other hand, LNPs are in an uncharged state at a neutral pH, such as when they are delivered *in vivo*, thus preventing immune system reaction. Once LNPs are internalized into target cells by endocytosis, they become positively charged again due to the lowering of the endosomal pH. This provokes the rupture of the endosome membrane enabling the release of the nucleic acids into the cytosol (Paunovska et al., 2022). Up to now, the efficient encapsulation into LNPs is limited to nucleic acids, which remain in the cytosol and cannot access the nucleus, after delivery to target cells. Since the persistence of nucleic acids in the cytosols is limited in time, LNPs allow for transient delivery of their cargo, overcoming the limitation related to the stable expression of the editing agents by viral vectors. Once injected into the bloodstream, LNPs are coated with ApoE lipoproteins that interact with the LDLR. Since LDLR is highly expressed by hepatocytes, LNPs efficiently accumulate in the liver (Paunovska et al., 2022). Not only the LNPs’ chemical composition but also the cargo ones have to be fine-tuned for efficient delivery. For example, RNA molecules have been optimized to increase their stability and decrease their immunogenic profile. Cas9 mRNAs have been modified by exploiting pseudouridine, 5-methylcytidine, and capping (Karikó et al., 2008; Mauer et al., 2017), while 2′OMe, 2′F, and phosphorothioate have been included in sgRNA in a specific order (Yin et al., 2017). All these improvements led to preclinical studies aimed at evaluating the potency of LNPs to deliver gene-editing agents to hepatocytes for the treatment of different disorders. The delivery of Cas9 mRNA together with sgRNA has been tested for the disruption of genes in the context of hereditary transthyretin amyloidosis or hereditary angioedema and led to the initiation of two clinical trials (Finn et al., 2018; Gillmore et al., 2021; Longhurst et al., 2024). LNPs have also been extensively tested for the delivery of BEs in mouse models of phenylketonuria (Brooks et al., 2023; Brooks et al., 2024) and alpha-1 antitrypsin deficiency (Packer et al., 2022) and in NHPs for the disruption of *PCSK9* (Musunuru et al., 2021; Rothgangl et al., 2021). Moreover, gene activators and gene silencers have been efficiently delivered to hepatocytes via LNPs to activate erythropoietin (Beyersdorf et al., 2022) and silence *PCSK9* (Cappelluti et al., 2024). Noticeably, combinations of different delivery methods have been exploited for genome editing applications involving the use of donor DNA. In this case, LNPs were used for the transient delivery of Cas9 and sgRNA, and AAVs for the nuclear delivery of donor DNA. This strategy has been tested in the context of hemophilia A and B, leading to the insertion in approximately 2%–3% of the target loci (Han et al., 2023; Lee et al., 2023). Notwithstanding the need for further optimizations to achieve higher efficiency, this combined delivery method is promising since it condensates the advantages of both LNPs and AAVs. Recently, LNPs have been further modified to allow their uptake in hepatocytes of patients lacking sufficient levels of LDLR, such as those with homozygous familial hypercholesterolemia. This outcome has been obtained by adding the N-acetylgalactosamine (GalNAc)-targeting ligand to the LNP, to generate GalNAc-LNP. This ligand is recognized by the asialoglycoprotein receptor (ASGPR), which is highly expressed in the liver and allows rapid endocytosis. In the work describing the strategy, Kasiewicz et al. demonstrated the ability of these GalNAc-LNP to deliver gene editing agents to hepatocytes of both wild-type or LDLR knock-out mice and NHPs (Kasiewicz et al., 2023).

### Virus-like particles

VLPs are derived mainly from retroviruses from which they maintain the envelope and the capsid, while the viral genome is substituted by the gene editing agents incorporated as RNA or protein (Lyu et al., 2020). In this way, VLPs can exploit the virus’s capacity to enter the cell while allowing transient delivery of the editors in the cell, reducing the risk of off-target events and immunogenicity. Retroviruses were selected since their immature particles are 100 nm-long in diameter, spherical, and relatively flexible, allowing the packaging of large cargo such as Cas9 or BEs (Raguram et al., 2022). The first tested VLPs were packaged with mRNA templates. However, without the addition of proper chemical mRNA modifications, this template is rapidly degraded once inside the cells, thus resulting in low editing efficiency. On the other hand, the most efficient strategy developed for *in vivo* applications is based on the packaging of proteins or RNPs (Figure 9E). To allow for their encapsulation, proteins or RNP have been fused to structural viral proteins, usually *gag*. Subsequently, the release of the cargo occurs during virion maturation by exploiting a cleavable peptide. Upon cell transduction, the VLP cargo is released inside the cytosol (Kaczmarczyk et al., 2011). The formation of the RNPs takes place during VLP production thanks to the expression of the sgRNA from an additional plasmid. Different configurations have been tested in cell lines or for *ex vivo* applications but just two of them achieved efficient editing in the liver. The first one is called “nanoblade” and contains SpCas9-RNP fused to murine leukemia virus (MLV) *gag*. Nanoblades were shown to achieve 10% of editing in the liver of HT1 mice (Mangeot et al., 2019). Other tools are engineered VLPs (eVLPs), based on Moloney MLV (MMLV), in which Cas9 or base editor-RNP are fused to the MMLV *gag* with an engineered linker peptide. eVLPs delivered *in vivo* to the liver achieved up to 63% editing efficiency to disrupt *PCSK9* gene (Banskota et al., 2022). eVLPs were also optimized to package PEs as RNP, but up to date they were only tested for *in vivo* editing of the retina (An et al., 2024). Despite the promise of eVLPs for efficient hepatic delivery, their immunogenic profile should be the object of careful evaluation since they are of viral origins.

## Liver-directed genome editing clinical trials

There are currently eight clinical trials employing *in vivo* liver-directed gene editing approaches (Table 2). The first one started in 2020 (Figure 2), sponsored by Intellia Therapeutics, for the treatment of Transthyretin Amyloidosis with polyneuropathy or cardiomyopathy (ClinicalTrials, 2023). This disease is caused by acquired or hereditary dominant mutations in the *TTR* gene and is characterized by the accumulation of the misfolded transthyretin in tissues. The genome editing treatment is based on the intravenous administration of LNPs carrying SpCas9 mRNA and sgRNA targeting the *TTR* gene in hepatocytes, to disrupt its expression and avoid the toxic TTR accumulation. Results published from the patients treated in this phase I clinical trial showed a significant and durable reduction in serum TTR, with no SAEs (Gillmore et al., 2021; Intellia Therapeutics, 2023). Those promising results led to the initiation of the first Phase III clinical trial for *in vivo* liver-directed gene editing, with more than 30 patients dosed so far (ClinicalTrials, 2024b; Intellia Therapeutics, 2024). The second clinical trial started in 2021, sponsored again by Intellia Therapeutics, for the treatment of Hereditary Angioedema (ClinicalTrials, 2024c). The disease is characterized by unpredictable swelling caused by mutations of the gene encoding for C1-inhibitor. This causes dysregulation of the complement contact activation pathway which can be restored by disrupting the kallikrein expression from the *KLKB1* gene. The treatment is again based on intravenous administration of LNPs carrying SpCas9 mRNA and sgRNA targeting the *KLKB1* gene in hepatocytes. The results of the first 10 treated patients were published in 2024, showing no major SAEs, a dose-dependent reduction in kallikrein plasma levels up to 95%, and an overall 80%–91% reduction in angioedema episodes (Longhurst et al., 2024). The study is now completed with 37 patients enrolled and Intellia is planning to start a Phase III study in the second half of 2024 (Intellia Therapeutics, 2024). The third study, sponsored by Verve Therapeutics, was the first to evaluate a base editing platform using an ABE to disrupt the expression of the *PCSK9* gene, to treat heterozygous familial hypercholesterolemia (ClinicalTrials, 2024d). The treatment is based on the intravenous administration of LNPs carrying ABE-mRNA and sgRNA targeting the *PCSK9* gene in hepatocytes. Thirteen patients were dosed with 4 different doses of LNPs, achieving an average 46% LDL-cholesterol reduction at a dose of 0.45 mg/kg. However, one of the patients treated with this LNP dose experienced an SAE, presenting with Grade 3 elevation of serum alanine aminotransferase (ALT) and drug-induced thrombocytopenia within the first 4 days after dosing. For this reason, the trial is currently on hold (Verve Therapeutics, 2024b). The sponsor hypothesized the SAE to be related to the LNP formulation exploited for the delivery. For this reason, a new clinical trial started in May 2024, by employing a new delivery method based on GalNAc-LNPs (ClinicalTrials, 2024e). Data on this trial will be released in 2025 (Verve Therapeutics, 2024a). At the end of 2023, CRISPR Therapeutics announced the starting of two Phase I clinical trials to treat cardiovascular diseases. The trials are based on LNPs delivering Cas9 mRNA and sgRNA targeting either ANGPTL3 or lipoprotein(a) in LDLR knock-out human livers (CRISPR Therapeutics, 2023). Up to date, no data about these trials have been released. Finally, In June 2024, Beam Therapeutics announced the dosing of the first patient in a clinical trial for Alpha-1 Antitrypsin Deficiency (Beam Therapeutics, 2024; ClinicalTrials, 2024f). A severe form of the disease is characterized by a point mutation in the *SERPINA1* gene, which causes an inefficient secretion of the alpha-1 antitrypsin protein that accumulates in hepatocytes. The treatment is based on the systemic administration of liver-targeting LNP carrying ABE-mRNA and sgRNA to correct the abovementioned point mutation. Other trials are supposed to start between the end of 2024 and the beginning of 2025. For instance, Intellia Therapeutics is developing an *in vivo* liver-directed CRISPR/Cas9-based therapy for treating Alpha-1 Antitrypsin Deficiency. The treatment exploits LNPs to deliver Cas9 mRNA and sgRNA, and an AAV to deliver the donor DNA encoding the AAT protein. The strategy is designed to insert the *AAT*-donor DNA into the albumin safe harbor locus (Intellia Therapeutics, 2024). Moreover, Verve Therapeutics is proposing another base editing program to inactivate the *ANGPTL3* gene in the liver for the treatment of homozygous familial hypercholesterolemia (Verve Therapeutics, 2024a). The rapid development of genome editing technologies from basic science tools to clinical-stage therapeutic products highlights their potential for the treatment of liver genetic diseases and opens the possibility for their application even to acquired diseases. As an example, Verve Therapeutics included acquired cardiovascular diseases in the company research pipeline (https://www.vervetx.com/our-programs/our-pipeline). However, several challenges remain to be addressed to increase editing efficiency, safety, and clinical translatability.

**TABLE 2:** Current status of Liver genome editing active clinical trials.

Disease indication	Genome editing strategy	Delivery method	Sponsor	Study type	References
Hereditary transthyretin amyloidosis with polyneuropathy, hereditary transthyretin amyloidosis with cardiomyopathy, or wild-type cardiomyopathy	*TTR* gene disruption	LNPs encapsulating SpCas9 mRNA + sgRNA	Intellia Therapeutics	Phase I	-ClinicalTrials.gov Identifier: NCT04601051 Gillmore et al. (2021)
				Phase III	-ClinicalTrials.gov Identifier: NCT06128629
Hereditary Angioedema	*KLKB1* gene disruption	LNPs encapsulating SpCas9 mRNA + sgRNA	Intellia Therapeutics	Phase I/II	-ClinicalTrials.gov Identifier: NCT05120830 Longhurst et al. ( 2024)
Heterozygous familial hypercholesterolemia, atherosclerotic cardiovascular disease	*PCSK9* gene disruption	LNPs encapsulating ABE mRNA + sgRNA	Verve Therapeutics Verve Therapeutics	Phase I	-ClinicalTrials.gov Identifier: NCT05398029
				Phase I	-ClinicalTrials.gov Identifier: NCT06164730
Cardiovascular diseases	*ANGPL3* gene disruption	LNPs encapsulating SpCas9 mRNA + sgRNA	CRISPR Therapeutics	Phase I	CRISPR Therapeutics (2023)
	Lipoprotein(a) disruption			Phase I	
Alpha-1 Antitrypsin Deficiency (AATD)	SERPINA1 gene correction	LNPs encapsulating ABE mRNA + sgRNA	Beam Therapeutics	Phase I/II	-ClinicalTrials.gov Identifier: NCT06389877

## Genome editing-related potential adverse events

When exploiting the CRISPR/Cas9 system with nucleases one major concern is related to the potential off-target effects that could lead to genomic alterations following DSB (Tao et al., 2023). Examples of genomic alterations are undesired indels, large deletions, or even chromosomal rearrangements with chromothripsis in both on- and off-target sites (Leibowitz et al., 2021; Tao et al., 2023). Over the years different methods have been developed to predict and detect off-target events. One of the most used *in silico* tools that allows the prediction of sequences with high similarity to the one recognized by the sgRNA is Cas-OFFinder (Bae et al., 2014). Other strategies exploit *in vitro* digestion of genomic DNA by the nuclease. One of the latest *in vitro* assays is CIRCLE-seq, based on the linearization and amplification of the DNA fragment containing a Cas9 cleavage site followed by its sequencing (Tsai et al., 2017). However, in both *in silico* and cell-free methods, the possibility of retrieving false positives is high, thus their results need validation. In this context, methods based on Cas activity directly inside the cells are more reliable. The most used method is GUIDE-seq, based on the insertion of short double-stranded oligodeoxynucleotides into the DSBs created by the nuclease. The regions with the insertion are then sequenced to identify the off-targets (Tsai et al., 2015). A limitation of this method is the requirement for cells that can be easily transfected. Other strategies have been developed to find larger deletions or translocations. For instance, chromosomal aberrations analysis by single targeted linker-mediated PCR sequencing (CAST-seq) is now extensively used because it can quantitatively calculate the frequency of those aberrations (Turchiano et al., 2021; Klermund et al., 2024). The major limitation of CAST-seq is the high amount of input genomes required to detect translocation events since those chromosomal aberrations are relatively rare events. However, the results obtained with all approaches need to be always experimentally validated by targeted sequencing of the putative off-target region or long-read sequencing of the chromosomal aberrations, to verify the off-target cleavage or to detect eventual technical bias (Tao et al., 2023; Torella et al., 2024). To reduce the off-targets, many high-fidelity Cas9 have been developed, among others HIFI Cas9 (Vakulskas et al., 2018) and EvoCas9 (Casini et al., 2018). In parallel, a careful selection of the optimal sgRNA has to be always performed to balance the cutting efficiency and the probability of off-target effects. When employing BEs, a major concern is to provoke unintended edits, in either the on-target or the off-target sites. The so-called “bystander edits” can occur on the on-target locus if in the spatial activity window of the deaminase are present nucleotides that can be modified other than the target one (Porto and Komor, 2023). BEs can present the same sgRNA-dependent off-target events as Cas9 but also sgRNA-independent off-targets (Porto and Komor, 2023). The sgRNA-dependent off-targets are due to the pairing of the gRNA on homologous loci and can be detected and solved using the same methods developed for Cas9, as described above. The sgRNA-independent off-targets can occur both at DNA and RNA levels, and they are complicated to detect and analyze. The methods exploited are genome-wide (Zuo et al., 2019) or transcriptome-wide sequencing (Grünewald et al., 2019). However, they are very expensive, and data retrieved from this kind of analysis require specific bioinformatics expertise to be analyzed. To reduce the sgRNA-independent off-targets, deaminases with a higher fidelity have been developed. However, the increase in specificity is often at the expense of reduced efficiency (Doman et al., 2020; Neugebauer et al., 2023). As Cas9 and BEs, also PEs can generate off-target editing caused by the recognition of homologous sequences by the sgRNA, which can be prevented by using high-fidelity Cas9 (Kim et al., 2020). The possibility of sgRNA-independent off-target of PEs has been investigated but without significant findings, at least so far (Gao et al., 2022). Nevertheless, it has been shown that PEs could eventually retro-transcribe part of the sgRNA scaffold, resulting in its potentially detrimental insertion at the edited site (Tao et al., 2022). Moreover, studies performed in HSCs have shown the introduction of chromosomal aberrations when using BEs and PEs, even if to a lower extent than Cas9. This is due to the usage of nCas9, which can, in some cases, induce the formation of a DSB (Fiumara et al., 2024). In the context of HSCs, it has also been demonstrated that all the different gene editing agents can cause an inflammatory response together with the activation of pathways that are detrimental to cell fitness, such as the p53-mediated DNA damage response (Schiroli et al., 2019; Fiumara et al., 2024). Many of these aspects have not yet been investigated for *in vivo* genome editing approaches directed to the liver. However, it is important to consider all the possible risks that in a clinical setting could give rise to a broad spectrum of adverse events, from allergic reactions to specific drug formulations to persistent tissue or organ damage leading to potential tumorigenesis. A way to limit potential toxicities is to transiently deliver the editing components, as obtained by the use of LNP-mRNA as previously described. However, even the potential toxicity related to the vehicle itself has to be evaluated. Regarding LNPs, important concerns were raised during the first trial from Verve Therapeutics, which was paused due to toxicity possibly related to the LNP formulation (Verve Therapeutics, 2024b). Concerning viral delivery vehicles, multi-year pre-clinical and clinical experience has demonstrated their therapeutic potential but also highlighted some related toxicities. Among these, the vector-induced immune responses can lead to the elimination of transduced cells or even to SAEs, especially when administering high vector doses (Shirley et al., 2020). Furthermore, many viral vectors present a non-null risk of genotoxicity due to vector integration (Baruteau et al., 2024). Recently, it has been demonstrated that AAVs generate insertions of concatemers in almost half of the edited cells when used as delivery vehicles for donor DNA (Suchy et al., 2024). The phenotypic variability that can derive from those insertions must be carefully evaluated. Another aspect to be considered before the treatment of patients is the pre-existing immunity either against the vehicle or the genome editing components. AAV vector immunity has a very high prevalence (30%–60%) in the population since wild-type AAV infection is very common (Weber, 2021). This is currently an exclusion criterion in most of the gene therapy clinical trials using those vectors for liver targeting. Moreover, it has been demonstrated that the presence of anti-Cas9 immunity is high in the population, however, it remains debated whether immune tolerance also occurs toward Cas9-derived antigens (Simhadri et al., 2018; Charlesworth et al., 2019). Since Cas9 is of bacterial origin, this aspect has to be taken into account when administering a gene editing product *in vivo* because it can cause an immune response against cells that are transduced/transfected (Li et al., 2020). In the latest trials, the presence of anti-Cas9 antibodies has been investigated before the administration of the genome editing product, but it has not been included among the exclusion criteria (Longhurst et al., 2024).

## Future perspectives

The future of gene editing looks bright. Clinical trials are generating very promising results and we are probably not far away from the approval of the first *in vivo* gene editing product. Those results are impressive considering that only around a decade has passed between the discovery of CRISPR/Cas9 and its approval in the clinic. For now, strategies that reached the clinic for liver-directed gene editing are based on Cas9 or BEs to disrupt a specific gene. Gene disruption is the simplest and most efficient modification that can be achieved so far. Efficient small genomic insertions have been obtained only for *ex vivo* applications, such as in the PEs-based program for the treatment of Chronic Granulomatous Disease by Prime Medicine (Prime Medicine, 2024). Likely, PE efficiencies still need to be improved to enable *in vivo* clinical applications. The introduction of large genomic insertions remains a major challenge. The development of a unique strategy based on the insertion of a corrective cDNA would be ideal to correct at once multiple gene mutations. In this context, positive results have been obtained by Intellia, exploiting the insertion of a corrective donor DNA for the treatment of Alpha-1 Antitrypsin Deficiency, leading to the initiation of a clinical trial (Intellia Therapeutics, 2024). Intellia is applying a similar genome editing strategy in hemophilia A and B pre-clinical studies. Other strategies aiming at introducing large insertions are under investigation. These are based on serine integrases, such as PASTE and PASSIGE methods described above, or on transposases, such as the FiCAT (find and cut-and-transfer) system. FiCAT exploits an engineered piggyBac transposase combined with Cas9 (Pallarès-Masmitjà et al., 2021), to achieve a transposase-mediated insertion of a donor DNA upon Cas9-induced DBS, bypassing the requirement of the TTAA motif at the insertion site. The system was tested *in vivo* but with low efficacy of the integration rate (Pallarès-Masmitjà et al., 2021). An interesting discovery to be mentioned was the CRISPR-associated transposase (CAST) bacterial system. Two different CAST types have been described, type V-K CAST (Strecker et al., 2019) and type I-F CAST (Klompe et al., 2019). Both are based on a nuclease-inactive Cas that, upon DNA binding *via* the sgRNA, is able to recruit the transposon that allows the site-specific insertion of large sequences. To date, those systems demonstrated very high integration capacity in the bacterial genome but a low integration rate in mammalian cells, suggesting the requirement of additional efforts to advance these tools (Tou et al., 2023; Lampe et al., 2024). Lastly, RNA editing technologies are emerging to avoid potential genotoxicity due to DNA editing. The most exploited system is based on Cas13a, which was tested to reduce gene expression by the site-specific cleavage of a target RNA (Abudayyeh et al., 2017). In addition, RNA-base editing technologies have been developed exploiting enzymes of the ADAR family which catalyze A-to-I editing. Those enzymes have been tested in mouse models of ornithine transcarbamylase (Katrekar et al., 2019) and mucopolysaccharidosis type I syndrome (Katrekar et al., 2022; Yi et al., 2022). However, the low *in vivo* efficiencies mandate for the need of further improvement to achieve therapeutic efficacy. In conclusion, all the scientific advancements obtained in the last years hold great promises for the beginning of an era in which liver-targeted genome editing could become a standard of care for both genetic and acquired diseases. Importantly, the rapid development of these genomic technologies has to be paralleled by a concomitant careful investigation of safety and ethical aspects related to their usage.
